# Effectiveness of App-Based Yoga of Immortals (YOI) Intervention for Insomnia in Asian Population during Pandemic Restrictions

**DOI:** 10.3390/ijerph18115706

**Published:** 2021-05-26

**Authors:** Renuka Tunuguntla, Hari Siva Gurunadha Rao Tunuguntla, Himanshu Kathuria, Sadhna Verma

**Affiliations:** 1 Hunterdon Medical Center, Department of Internal Medicine, 121 Route 31, Suite 1000, Fleminton, NJ 08822, USA; 2 Rutgers, State University of New Jersey, Robert Wood Johnson Medical School & Robert Wood Johnson University Hospital, 1, RWJ Place, New Brunswick, NJ 08901, USA; tunuguha@rwjms.rutgers.edu; 3 Department of Pharmacy, National University of Singapore, Singapore 117543, Singapore; himanshukathuria01@u.nus.edu; 4 The Cincinnati Veterans Administration Hospital, University of Cincinnati College of Medicine, 234 Goodman Street, Cincinnati, OH 45267, USA; vermasm@ucmail.uc.edu

**Keywords:** sleep disorders, yoga, exercise, healthcare delivery, meditation

## Abstract

The coronavirus disease 2019 (COVID-19) pandemic created significant psychological challenges worldwide, including stress, emotional distress, and insomnia. In addition, social distancing, travel restrictions, and spread of disease have resulted in unique challenges, creating barriers to healthcare access. Compared to the rate prior to the COVID-19 pandemic, a significant increase in clinical insomnia rates have been reported. With well-known limitations of currently established treatments (e.g., cognitive behavioral therapy-insomnia (CBT-I), pharmacotherapy), there is a need to explore other effective and safe treatment modalities to treat insomnia, especially those that can be used remotely. The purpose of this study is to assess the effectiveness of app-based intervention to treat insomnia in the current era of the COVID-19 pandemic (using the Yoga of Immortals (YOI) app). This prospective cohort study was approved by the Institutional Review Board. All participants in this study were asked to complete an online survey including demographic data and validated Insomnia Severity Index (ISI) at baseline (15 May 2020), 4 weeks, and 8 weeks after starting the YOI intervention. Survey data was exported using Microsoft Excel. Statistical analysis was done using the GraphPad Prism 8. YOI intervention significantly improved the mean ISI scores in all categories of insomnia (severe, moderate, and subthreshold) at each follow-up (*p* ≤ 0.0001). The improvement was significant among all age groups and in both genders. In our study, YOI was a novel and effective intervention for improving insomnia symptoms and may be a new addition to the armamentarium of insomnia management. Being app-based, this has potential wider applicability, especially during the current COVID-19 pandemic.

## 1. Introduction

The coronavirus disease 2019 (COVID-19) pandemic has brought unprecedented changes, affecting everyday life in many different ways, including worries about health, employment, finances, and the challenges of combining work and family obligations [1]. Social distancing, lockdowns, dwindling economy, and unemployment are posing major challenges all over the world, and creating stressful circumstances and health risks that can have a major impact on nighttime sleep, at a time when healthy sleep is of paramount importance in order to cope and adapt with this crisis and uncertainty about the future [1].

Insomnia is the most common sleep-related complaint and the second most common overall complaint (after pain) reported in primary care practice, with about 30–50% of adults reporting problems with sleep in a given year [2]. Based on many population studies, one-third of adults have frequent trouble falling asleep, staying asleep, or overall poor sleep quality [3]. In addition to the substantial suffering of individuals with insomnia, the disorder also causes high societal costs because of sick leave, use of healthcare, and lost productivity. Insomnia is associated with, and is a suspected contributing factor to, conditions such as cardiac disease, diabetes, anxiety, and depression [4]. While investigating the early impact of the COVID-19 pandemic on sleep and psychological symptoms in 5641 Chinese adults in mid-February 2020, Lin et al. noted very high rates of clinically significant insomnia (20%) and acute stress (15.8%) [5].

Insomnia is associated with several adverse health outcomes, such as poor physical health, poor mental health, including symptoms of anxiety and depression, and decreased quality of life [6,7]. Cognitive behavioral therapy for insomnia (CBT-I), traditionally used to treat chronic insomnia, has recently been used to treat acute insomnia secondary to the situational changes from acute stress [8,9]. Although CBT-I is traditionally delivered in face-to-face individuals or group settings, remote delivery online or by telephone has also been used, albeit with less robust supporting data. Though CBT-I is an effective and recommended first line treatment modality, it needs trained providers. Usually, there is a delay in therapeutic response, which limits its use and acceptance. Due to these limitations, most people choose pharmacological treatment for insomnia. Commonly used medications are short or intermediate acting benzodiazepine receptor agonists that have significant side effects. Notable side effects are drowsiness, fatigue, confusion, ataxia, falls, hip fractures, anterograde amnesia, anxiety, complex sleep related behaviors, such as sleepwalking, and serious adverse effects, including dependence, with grave consequences and economic burdens [10]. Currently, an estimated USD 30–107 billion is annually spent on insomnia management in the U.S. [11,12]. In addition, insomnia has been reported to result in an annual loss of workplace productivity to an estimated USD 63.2 billion [13]. Therefore, there is an urgent need to explore novel and innovative treatment modalities that are easily available with the least (or no) side effects and are cost-effective, without the need for in-person contact.

Yoga of Immortals (YOI) is a comprehensive program developed by ShivYog, an organization that teaches specific practices based on ancient yogic teachings [14,15]. Much of today’s yoga, meditation, and mindfulness practices are derived from these ancient yogic teachings [16]. Although YOI has been in practice in the Eastern hemisphere for centuries with many health benefits, it was only taught through in-person meetings to a very few individuals. Hence, no published outcome data are available to date. A structured YOI program was recently developed for a mobile platform—a platform that is increasingly being utilized and accepted in today’s technological society. This study investigates whether the app-based YOI intervention effectively treats insomnia symptoms, especially in the background of a global pandemic that has caused immense population distress and simultaneous disruption of access to care.

## 2. Materials and Methods

### 2.1. Study Population

Study participants were subscribers of the YOI app from all over the world; they were willing to participate in the study and complete the surveys. Baseline survey was sent on 15 May 2020) to all subscribers of the app, which included a demographic questionnaire and validated Insomnia Severity Index (ISI) questionnaire (Figure S1) [17]. The participants were then invited to complete the YOI intervention. Follow-up surveys were sent at 4 and 8 weeks. One week following the surveys, reminder emails were sent to all participants, encouraging them to complete the survey (if not already done).

### 2.2. Inclusion Criteria

The inclusion criteria were age ≥18 years, provide informed consent, and complete baseline, 4-week, and 8-week survey forms.

### 2.3. Study Design

**Figure 1:**
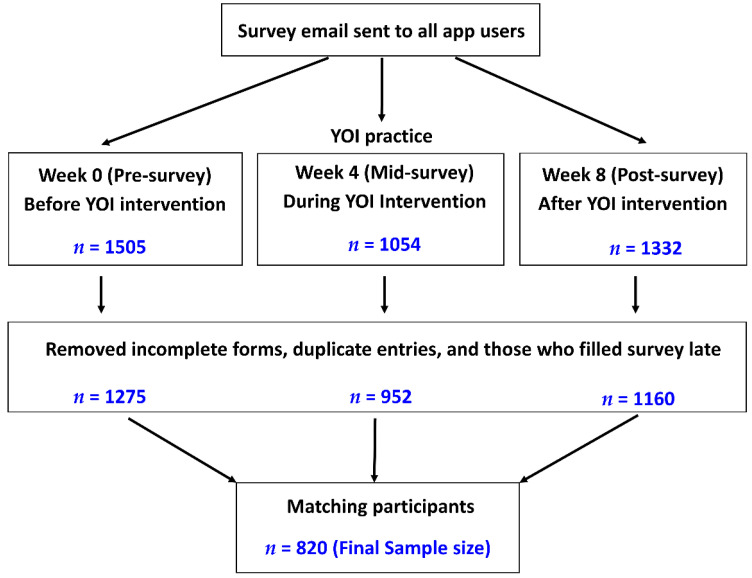
Outline of the study process, participants’ selection criteria, and selection of the final number of participants for data analysis.

Initially, 1505 participants completed the baseline survey, out of which 1275 remained after excluding incomplete and duplicate surveys. At 4 weeks, 952 participants completed the survey and fulfilled the criteria, and at 8 weeks, 1160 fulfilled the criteria. Therefore, only those participants who completed baseline (pre-survey), 4-week (mid-survey), and 8-week (post-survey) surveys (matching participants) were included in the final study analysis (Figure 1).

### 2.4. Study Approval

The study was approved by the Institutional Review Board (IRB), University of Cincinnati, Cincinnati, Ohio.

### 2.5. YOI Intervention

This was a unique intervention in video and audio format that combined specific breathing exercises with whole body exercises, yogic postures, and meditation, synchronized with sound therapy (chants) [14,15]. It consisted of weekly protocols, with one or two daily sessions (morning and evening) (Table S1). Meditation focused on energy centers and the pineal gland. The protocols were progressive, building on the earlier one, and changed weekly.

### 2.6. Assessment Scales

The Insomnia Severity Index (ISI), a validated scale, was used to assess insomnia symptoms in our study [17]. This instrument poses seven questions to assess current (i.e., preceding 2 weeks) sleep characteristics. Items are rated on a five-point Likert scale (‘0’ representing none or not at all, and ‘4’ representing very much) [18]. Total scores range from 0 to 28, with higher combined scores indicating worse insomnia severity [17]. Based on total scores, there are four categories, 0–7, no clinically significant insomnia; 8–14, subthreshold insomnia; 15–21, clinically significant insomnia (moderate); and 22–28, clinically significant insomnia (severe).

### 2.7. Statistical Analysis

The survey responses were exported, organized, and processed using Microsoft Excel. Human verification and attention checks were implemented throughout the survey to ensure data quality. Statistical analysis was conducted using the GraphPad Prism 8 (Graph Pad Software, Inc., San Diego, CA, USA). The demographics are shown in numbers and percentages. The descriptive statistics are shown in number, difference (δ), and percentage difference (%δ). The comparative statistics for mean scores for different categories were conducted using analysis of variance (ANOVA) with post hoc test. One-way ANOVA was used for unmatched data, and one-way repeated measures ANOVA was used for matched data. For both tests, multiple comparisons were made using Tukey’s test. The *p*-value ≤ 0.05 and 95% confidence interval were considered as statistically significant. The baseline score of participants served as the control for comparison.

## 3. Results

**Figure 2:**
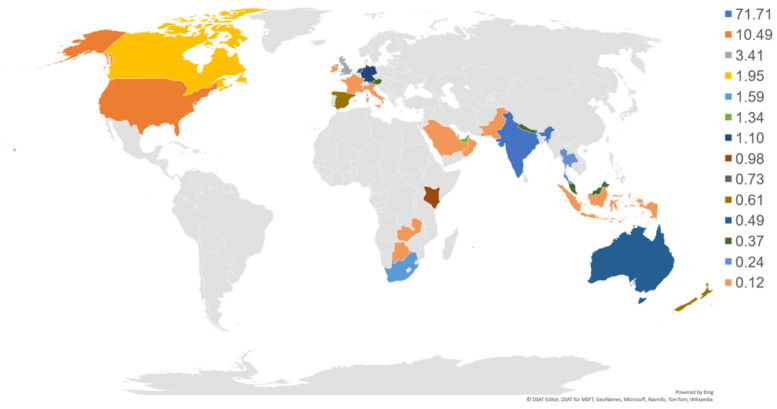
Graphical distribution (%) of participants in YOI intervention across nations. *n* = 820.

The demographic details categorized into age, gender, educational status, race and ethnicity, occupation, and associated psychiatric disorders are shown in Table 1. The maximum percentage of participants were in the age group of 26–47 (61.8%), followed by 48–58 (23.0%), 59–69 (8%), and 18–25 (6.3%). The percentage of participants >70 was less than 1%. The study participants had more females (53.8%) than males (46.1%). The highest qualification ranked from school level to Ph.D. level. Geographically, participants were from 14 different countries; however, the maximum number of participants were of Asian race (88.4%) (Figure 2).

**Figure 3:**
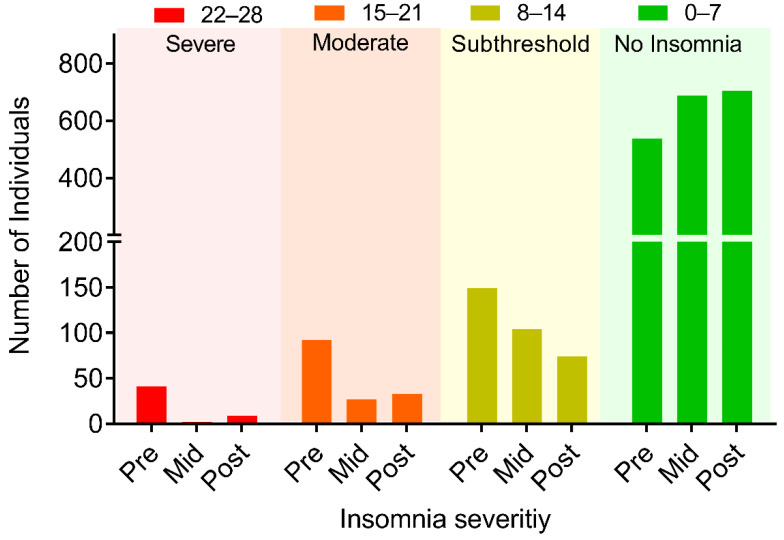
Changes in the number of individuals based on different insomnia severity categories before intervention (Pre), during the intervention (Mid, 4 weeks), and post-intervention (post, 8 weeks). *n* = 820.

Based on pre-survey (week 0) questionnaires, 41 (5%) participants had severe insomnia (SI), 92 (11.22%) had moderate insomnia (MI), 149 (18.17%) had subthreshold insomnia (STI) (Table 2 and Figure 3). Following YOI intervention, a marked reduction in the mean ISI score was noted in all categories, 23.88 ± 0.25 to 10.46 ± 1.27 in the severe group; 17.71 ± 0.21 to 6.51 ± 0.64 in the moderate group, and 10.87 ± 0.17 to 4.95 ± 0.38 in the subthreshold group (Table 2).

At baseline, 41 participants had severe insomnia; 92, moderate insomnia; and 149 had subthreshold insomnia. At 8 weeks, these numbers were reduced to 9 in the severe group, 33 in the moderate group, and 74 in the subthreshold group, with an increase in the number of participants in the ‘no clinically significant insomnia’ category (537 at baseline; increased to 703 at 8 weeks). Compared to baseline, in the severe group, there was 92.68% reduction in the number of participants at 4 weeks and 78.05% reduction at 8 weeks. Likewise, the number of participants with moderate insomnia reduced by 70.65% at 4 weeks and 64.13% at 8 weeks. The number of participants with subthreshold insomnia reduced by 30.87% at 4 weeks and further reduced by 50.34% at 8 weeks (Table 3). On the contrary, the number of participants with ‘no clinically significant insomnia’ increased by 27.75% at 4 weeks and 30.91% at 8 weeks (Table 3), indicating that those participants in the severe, moderate, and subthreshold categories moved into the ‘no clinically significant insomnia’ category after YOI intervention.

**Figure 4:**
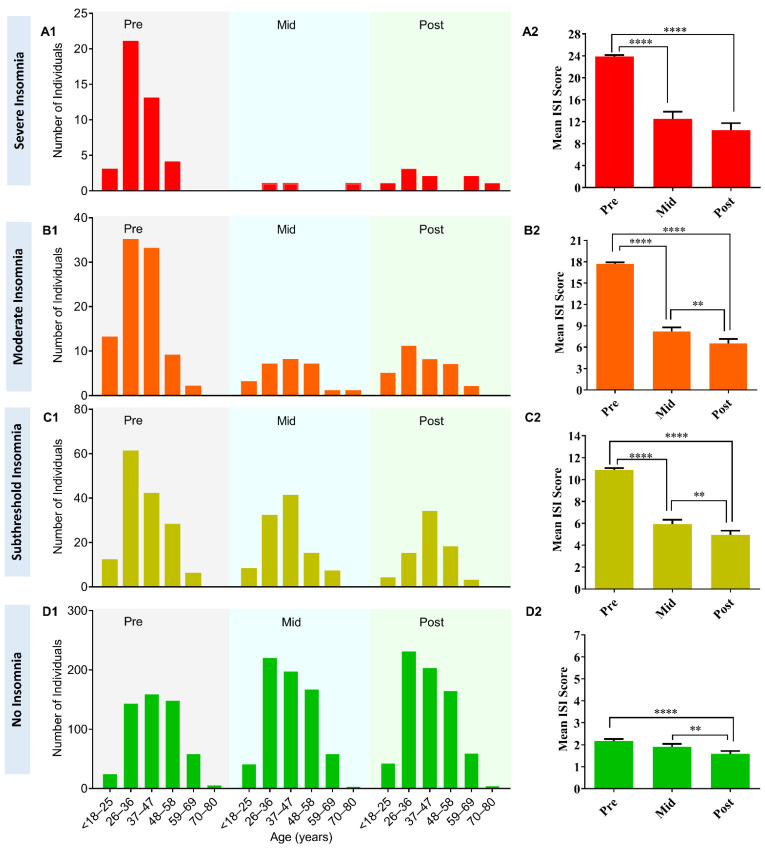
(**A1**–**D1**) Change in the number of individuals with YOI intervention among different age groups and different insomnia severity categories. Before YOI intervention (Pre), during YOI intervention (Mid, week 4), and post YOI intervention (Post, week 8). (**A2**–**D2**) Change in the mean ISI score with YOI intervention for different categories of insomnia. Before YOI intervention (Pre), during YOI intervention (Mid, week 4), and post YOI intervention (Post, week 8). Error bars are standard error of the mean (SEM). One-way repeated measures ANOVA with multiple comparisons using Tukey’s test was used for comparison with 95% confidence interval. ** *p* ≤ 0.01, **** *p* ≤ 0.0001.

When we analyzed the effect of YOI intervention in different age groups, there was a reduction in the number of participants with severe insomnia in all age groups except 59–69 years and 70–80 years. There was no one at baseline in the 59–69 age group, but two at 8 weeks. This may be due to the limited number of participants in these age groups. Likewise, there was no one at baseline in the 70–80 age group, but one at 8 weeks. In the moderate insomnia category, there was, again, a reduction in the number of participants in all age groups at 8 weeks, except for 59–69 years and 70–80 years. In the 59–69 age group, there were two participants at baseline, which remained the same at 8 weeks. In the subthreshold insomnia category, there was a reduction in the number of participants among all age groups at 8 weeks (Table 3; Figure 4, A1–D1).

**Figure 5:**
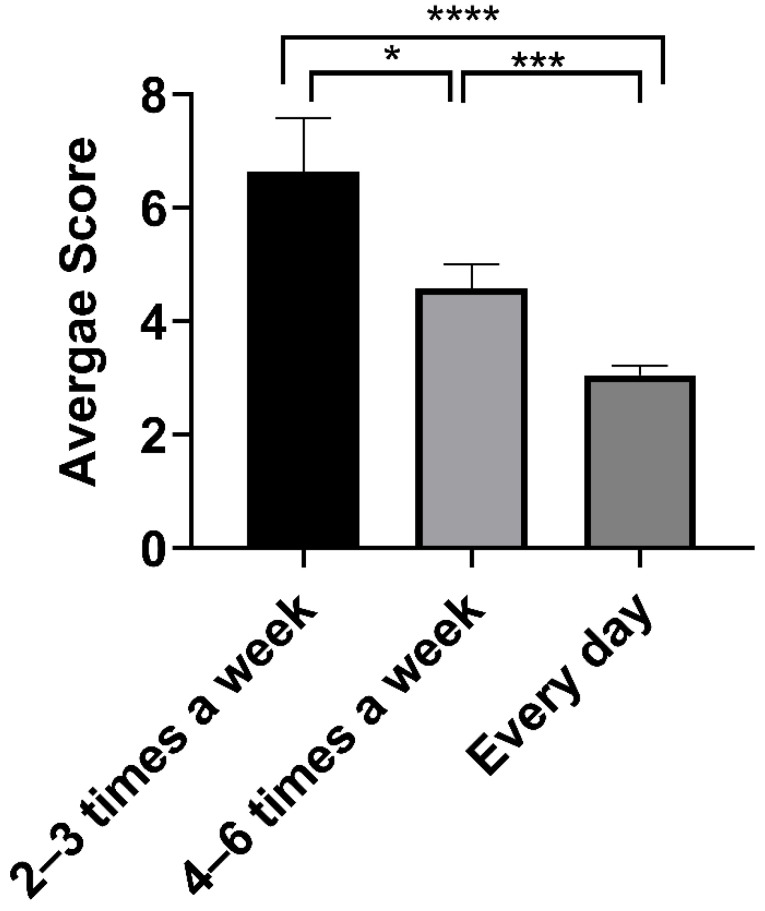
Mean ISI score for all participants with different frequency of YOI practice. Error bars are standard error of the mean (SEM). One-way ANOVA with multiple comparisons using Tukey’s test was used for comparison with 95% confidence interval. * *p* ≤ 0.05, *** *p* ≤ 0.001, ***** *p* ≤ 0.0001.

Although the participants were instructed to practice the YOI daily, it is conceivable that not everyone can practice every day due to personal commitments and other constraints. Therefore, we studied the effect of the frequency of YOI practice on the insomnia scores and noted that most participants (79.4%) practiced the YOI daily; 15.98% practiced 4–6 times per week, while 4.63% practiced 2–3 times per week. The higher percentage of participants with daily practice could be a sign of participant acceptance of YOI intervention, which could be due to benefits received to participants through daily practice. The mean ISI score was lowest for the participants who practiced the YOI everyday (Table 4; Figure 5). The scores for daily practitioners were one-half (3.05 ± 0.16) compared to those who practiced YOI 2–3 times (6.63 ± 0.95), or 4–6 times per week 4.58 ± 0.42. This suggests that participants who practiced YOI daily had better improvement than those who did not (Figure 5).

**Figure 6:**
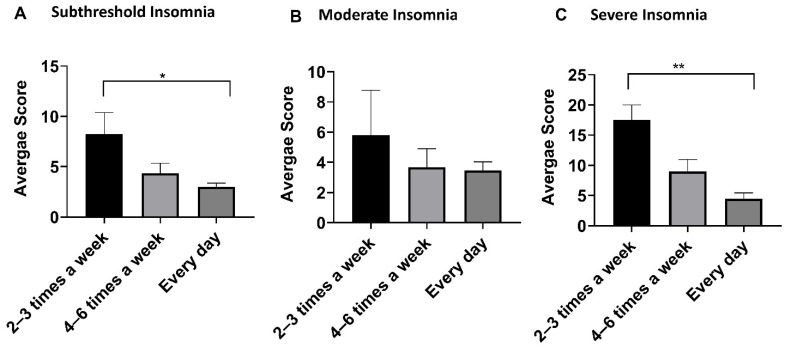
Mean insomnia score of different insomnia categories with different frequency of YOI practice. Error bars are Standard Error of mean (SEM). One-way ANOVA with multiple comparisons using Tukey’s test was used for comparison with 95% Confidence interval. * *p* ≤ 0.05, ** *p* ≤ 0.01.

We also compared the effect of the frequency of YOI practice in different insomnia categories (Table 5; Figure 6). We observed a decreasing trend in mean ISI scores with an increase in the frequency of YOI practice. There was a statistically significant difference in severe and subthreshold insomnia categories. Those practicing YOI intervention every day had the lowest mean score than those practicing 4–6 times, followed by the highest average score for those practicing 2–3 times a week.

Mean ISI scores were also assessed in participants with self-reported psychiatric disorders (Table 6). One hundred fifteen (14.02%) participants had psychiatric disorders, out of which 53 (46.09%) had more than one psychiatric disorder. In the latter group, the most commonly found conditions were major depression, generalized anxiety disorder (GAD), and eating disorders. After YOI intervention, we found a significant reduction in the mean ISI score in the multiple disorder group (13.53 ± 1.16 to 7.28 ± 0.97, *p* ≤ 0.0001)**.** The other group with a significant reduction in the mean ISI score was GAD (10.96 ± 1.60 to 5.26 ± 1.00, *p* ≤ 0.01). Compared to baseline, the reduction in the mean scores at 8 weeks was ~50% in both.

## 4. Discussion

Sleep is a very important factor affecting the health of an individual, just like nutrition. It is a basic requirement for the normal functioning of metabolic, endocrine, neurological, and cognitive functions; it is vital for one’s health and general well-being. Currently, cognitive behavioral therapy (CBT-I) and pharmacological intervention constitute the mainstay treatments of insomnia. Clinical practice guidelines published by the American College of Physicians (2016) recommended CBT-I as the initial treatment for insomnia [11]. Although CBT-I is considered the treatment of choice, the availability of trained personnel is the limiting factor for the effective implementation of a structured CBT-I program. In addition, CBT-I is not widely used, partly due to the cost of treatment by trained CBT-I providers and lack of insurance coverage for these services [19]. Furthermore, psychological and behavioral interventions are complex, have various protocols, and may have varying degrees of success for different individuals [20]. These issues with CBT-I carry even greater significance in the current setting of the COVID 19 pandemic.

According to the clinical practice guidelines published by the American College of Physicians (2016), CBT-I has been documented to result in a mean difference in the ISI score of −4.78 (95% CI, −6.45 to −3.11) compared to the inactive control [11]. Our study noted a 13.42 point reduction in the ISI score in the severe group, an 11.2 point reduction in the moderate group, and a 5.92 point reduction in the subthreshold group (Table 2) at 8 weeks following YOI intervention. Besides, the YOI mobile app-based intervention in the current study effectively reduced insomnia symptom severity at 4 weeks. It was maintained at 8 weeks with a statistically significant decrease in the mean ISI scores in all insomnia categories (Table 2). YOI intervention also showed marked improvement in the ISI scores among most age groups (Table 3)). We also assessed the effect of frequency of YOI practice on the outcomes and found a decreasing trend in the mean ISI scores with increasing frequency of practice among all insomnia categories (Table 5; Figure 6).

Regarding pharmacotherapy of insomnia, over the counter (OTC) sleep aids (e.g., diphenhydramine) are often used to manage sleeping difficulties, despite evidence suggesting such a practice may be risky. Prescription drug therapy includes benzodiazepines, non-benzodiazepine hypnotics, orexin receptor antagonist, the melatonin receptor agonist, ramelteon; the antidepressant, doxepin; and melatonin. Most prescription insomnia medications are either recommended on a limited basis or not recommended because of adverse outcomes. Observational studies have shown that hypnotic drugs may be associated with serious adverse effects, such as dementia, serious injury, and fractures [21,22].

A systematic evidence review of pharmacological therapies published by the American College of Physicians showed that eszopiclone, a non-benzodiazepine hypnotic, improved insomnia with a 4.6 point reduction in the ISI score [11]. Another agent, suvorexant, an orexin receptor antagonist, resulted in a 1.2 point reduction in the ISI score. However, a much higher reduction in the ISI scores was observed in our study using YOI intervention, as described above, in the comparison of our data with CBT-I.

Complementary and alternative therapies, such as Chinese herbal medicine, and acupuncture have also been used to treat insomnia. However, there is a paucity of good quality evidence on these therapies in insomnia. For example, Yin et al. showed improvement in the ISI score with acupuncture at 2 and 4 weeks post-treatment [23]. However, this is in-person treatment, and it requires the availability of a qualified and certified acupuncturist. In contrast, YOI is app-based and does not require an in-person visit.

Mind-body therapies (MBTs), including meditation, Tai chi, Qigong, and yoga, have been used to treat insomnia. These therapies have demonstrated some benefits in randomized control trials for insomnia and depression [24,25,26]. However, in a systematic review and meta-analysis that included 10 studies with 926 participants using ISI, Wang et al. did not find significant reduction in the severity of insomnia symptoms (effect size:−0.26; 95% CI: −0.60 to −0.09; *p* = 0.142) using mind–body therapies (MBTs) [27]. Although YOI utilizes certain meditative movements and breath regulation and exercises, it is fundamentally different from Tai chi and Qigong, and uniquely connects the individual to the universal energy source.

Beneficial effects of seated meditation, breathing practices, and yoga asana practices are associated with a reduction in sympathetic nervous system activation. This is proven by parallel reductions in catecholamines epinephrine and norepinephrine levels [28,29], reducing the stress, thereby improving insomnia. Normal healthy sleep, regulation of circadian rhythm, and maintenance of internal biological clock are dependent on melatonin, a hormone secreted in the pineal gland [30]. Meditation and yoga have been shown to enhance the activity of melatonin, thereby improving insomnia [28].

In our study, participants were from all over the world, spanning 14 countries in different continents, different ethnic groups, educational and employment statuses, and included both genders. Therefore, we feel the results of our study are mostly generalizable; however, most of the participants (~88%) were Asian in this study. To our knowledge, ours is the first known study of YOI intervention in participants with self-reported insomnia.

## 5. Limitations

One of the limitations of our study is that only participants with self-reported insomnia symptoms were included, and we did not differentiate acute from chronic insomnia. There was also no separate control group in the study. Another limitation could be selection bias, as this intervention may apply to only certain traits inclined towards these practices. Participant connection to the app may have an indirect beneficial effect towards alleviating the loneliness and social isolation possibly contributing to insomnia in some.

## 6. Conclusions

This is the first study reporting the efficacy of Yoga of Immortals (YOI) intervention (app-based) in insomnia. YOI reduced mean ISI scores in all categories of insomnia among most age groups in both genders. YOI also improved sleep in subjects with associated psychiatric conditions. YOI offers a novel, effective, and promising solution to remotely treat insomnia symptoms without the risks of pharmacotherapy and limitations of cognitive based therapy (CBT-I). YOI can be used as a stand-alone option or adjunctive treatment of insomnia, and may be used in healthy individuals to prevent insomnia.

## Figures and Tables

**Table 1:** Demographic details of the study population shown in numbers and percentages. *n* = 820.

Categories	Count	%
Age		
18–25	52	6.3
26–36	260	31.7
37–47	247	30.1
48–58	189	23.0
59–69	66	8.0
70–80	5	0.6
>80	1	0.1
Gender		
Female	441	53.8
Male	378	46.1
Prefer Not to Say	1	0.12
Highest Degree		
High School or Lower	111	13.5
Bachelor’s Degree	385	47.0
Master’s Degree	267	32.6
Medical Doctor	37	4.5
Ph.D.	20	2.4
Race and Ethnicity		
American Indian or Alaska Native	5	0.61
Asian	725	88.4
Black or African American	1	0.12
Native Hawaiian or Other Pacific Islander	1	0.12
White	7	0.85
Other	81	9.88
Occupation		
Student	49	5.98
Employed	652	79.51
Unemployed	119	14.51
Psychiatric Disorders		
Multiple Disorders ^#^	53	6.46
Generalized Anxiety Disorder	23	2.80
Major Depression	14	1.71
Eating Disorder	13	1.59
Post-Traumatic Stress Disorder	5	0.61
Psychotic Disorders (Including Schizophrenia)	3	0.37
Bipolar Disorder	2	0.24
Obsessive Compulsive Disorder	2	0.24
None of the Above	705	85.98

^#^ Participants who reported more than one disorder are considered in the multiple disorders category.

**Table 2:** Effect of YOI intervention on mean ISI score among insomnia categories. Values are shown in mean ± SEM. One-way repeated measures ANOVA with multiple comparisons using Tukey’s test was used for comparison with 95% confidence interval.

Insomnia Severity	Pre	Mid	Post
SI (*n* = 41)	23.88 ± 0.25	12.54 ± 1.28 ****	10.46 ± 1.27 ****
MI (*n* = 92)	17.71 ± 0.21	8.21 ± 0.57 ****	6.51 ± 0.64 ****
STI (*n* = 149)	10.87 ± 0.17	5.94 ± 0.39 ****	4.95 ± 0.38 ****
NI (*n* = 538)	2.16 ± 0.10	1.91 ± 0.13	1.58 ± 0.13 ****

SI: severe insomnia; MI: moderate insomnia; STI: subthreshold insomnia; NI: no clinically significant insomnia. ‘*’ is significance level compared to pre, where, **** *p* ≤ 0.0001.

**Table 3:** Effect of YOI intervention on insomnia severity with age. *n* = 820.

Age (Years)	18–25	26–36	37–47	48–58	59–69	70–80	Total	δ	% δ
Number of Severe Insomnia (SI)									
Pre	3	21	13	4	0	0	41	NA	NA
Mid	0	1	1	0	0	1	3	−38	−92.68
Post	1	3	2	0	2	1	9	−32	−78.05
Number of Moderate Insomnia (MI)									
Pre	13	35	33	9	2	0	92	NA	NA
Mid	3	7	8	7	1	1	27	−38	−70.65
Post	5	11	8	7	2	0	33	−32	−64.13
Number Subthreshold Insomnia (STI)									
Pre	12	61	42	28	6	0	149	NA	NA
Mid	8	32	41	15	7	0	103	−46	−30.87
Post	4	15	34	18	3	0	74	−75	−50.34
Number of No clinically significant Insomnia (NI)									
Pre	24	143	159	148	58	5	537	NA	NA
Mid	41	220	197	167	58	3	686	149	27.75
Post	42	231	203	164	59	4	703	166	30.91

SI: severe insomnia (22–28); MI: moderate insomnia (15–21); STI: subthreshold insomnia (8–14); NI: no clinically significant insomnia (0–7); NA: not applicable; δ: difference in numbers between pre and mid or pre and post; δ: percentage difference in numbers between pre and mid or pre and post based to baseline score.

**Table 4:** Effect of YOI practice on mean ISI score of all participants. *n* = 820.

YOI Practice	% Participants	Mean ISI Score
2–3 times a week (*n* = 38)	4.63	6.63 ± 0.95 ****
4–6 times a week (*n* = 131)	15.98	4.58 ± 0.42 ***
Every day (*n* = 651)	79.39	3.05 ± 0.16

‘*’ is significance level in comparison to everyday practice of YOI, where *** *p* ≤ 0.001, **** *p* ≤ 0.0001. ISI scores are depicted as mean ± SEM.

**Table 5:** Effect of YOI practice on mean ISI score among different categories of insomnia.

YOI Practice	SI	MI	STI
2–3 times a week	17.50 ± 2.50	5.80 ± 2.97	8.25 ± 2.14
4–6 times a week	9.00 ± 1.98	3.67 ± 1.24	4.35 ± 1.02
Every day	4.48 ± 0.97 **	3.47 ± 0.57	3.00 ± 0.37 *

‘*’ is significance level compared to the everyday practice of YOI, where * *p* ≤ 0.05, ** *p* ≤ 0.01. ISI scores are depicted as mean ± SEM.

**Table 6:** Changes in mean ISI scores in participants with different psychiatric disorders (*n* = 115). One-way repeated measures ANOVA with multiple comparisons using Tukey’s test was used for comparison with 95% confidence interval.

Self-Reported Psychiatric Disorders	Pre	Mid	Post
**Multiple disorders ^#^ (*n* = 53)**	13.53 ± 1.16	8.38 ± 0.93 ****	7.28 ± 0.97 ****
GAD (*n* = 23)	10.96 ±1.60	5.48 ± 0.98 ***	5.26 ± 1.00 **
MD (*n* = 14)	6.79 ±1.52	4.21 ± 1.48	4.00 ±1.36
ED (*n* = 13)	7.69 ± 2.53	4.15 ± 1.81	2.46 ± 1.17
PTSD (*n* = 5)	5.80 ± 2.35	5.80 ± 3.37	2.20 ± 1.11
PD (*n* = 3)	11.67 ± 6.01	8.33 ± 4.91	5.67 ± 3.41
BPD (*n* = 2)	4.00 ±3.00	4.00 ± 0.00	4.5 ± 0.35
OCD (*n* = 2)	23.00 ± 4.00	14.50 ± 6.50	14.5 ± 8.13

GAD: generalized anxiety disorder; MD: major depression; ED: Eating disorder; PTSD: Post traumatic stress disorder; PD: psychotic disorder (including Schizophrenia); BPD: bipolar disorder; OCD: obsessive compulsive disorder. ‘*’ is significance level compared to pre, where ** *p* ≤ 0.01, *** *p* ≤ 0.001, **** *p* ≤ 0.0001. ^#^ Participants who reported more than one disorder are considered in the multiple disorders category.

## Data Availability

The data presented in this study are available on request from the corresponding author. The data are not publicly available due to Ethical Concerns.

## Supplementary Materials

The following are available online at https://www.mdpi.com/article/10.3390/ijerph18115706/s1, Figure S1: Insomnia Severity Index questionnaires, Table S1: YOI protocol timings.

## Footnotes

This study was performed by a team of physicians and researchers with an interest in scientific research to validate the efficacies of complementary and alternative treatment modalities. The study attempted to verify the efficacy of Yoga of Immortals as an innovative and novel intervention strategy for insomnia.
