# Supplementary material for: Effectiveness of App-Based Yoga of Immortals (YOI) Intervention for Insomnia in Asian Population during Pandemic Restrictions
Source: Int J Environ Res Public Health. 2021 May 26;18(11):5706. doi: 10.3390/ijerph18115706 (PMC8199123; doi:10.3390/ijerph18115706)
Supplement: Supplementary file 1 [file ijerph-18-05706-s001.zip › ijerph-1219651-supplementary.pdf]

Supplementary Information

S1. Insomnia Severity Index Questionaries

Name: ______________________________ **Date:** ________________________

1. Please rate the current (i.e., last 2 weeks) **SEVERITY** of your insomnia problem(s).

	None	Mild	Moderate	Severe	Very
Difficulty falling asleep:	0	1	2	3	4
Difficulty staying asleep:	0	1	2	3	4
Problem waking up too early:	0	1	2	3	4

2. How **SATISFIED**/dissatisfied are you with your current sleep pattern?

Very Satisfied				Very Dissatisfied
0	1	2	3	4

3. To what extent do you consider your sleep problem to **INTERFERE** with your daily functioning (e.g. daytime fatigue, ability to function at work/daily chores, concentration, memory, mood, etc.).

Not at all Interfering	A Little	Somewhat	Much	Very Much Interfering
0	1	2	3	4

4. How **NOTICEABLE** to others do you think your sleeping problem is in terms of impairing the quality of your life?

Not at all Noticeable	Barely	Somewhat	Much	Very Much Noticeable
0	1	2	3	4

5. How **WORRIED**/distressed are you about your current sleep problem?

Not at all	A Little	Somewhat	Much	Very Much
0	1	2	3	4

Guidelines for Scoring/Interpretation:

Add scores for all seven items (1a+1b+1c+ 2+3+4+5) = _____

Total score ranges from 0-28

0-7 = No clinically significant insomnia

8-14 = Subthreshold insomnia

15-21 = Clinical insomnia (moderate severity)

22-28 = Clinical insomnia (severe)

Figure S1: Insomnia Severity index questionaries that were used in the survey. Reprinted (adapted) with permission from [1]. Copyright (2021) Elsevier.

S2. YOI protocol timings

Table S1. YOI protocols timings including instruction video and practice video/audio

Time	Instruction video time (min)	Practice video/audio time (min)
Week 0	None	None
Week 1	10	34
Week 2	8	34
Week 3	8	35
Week 4	10	35 (Morning), 45 (Evening)
Week 5	14	37 (Morning), 37 (Evening)
Week 6	18	33 (Morning), 36 (Evening)
Week 7	13	36 (Morning), 31 (Evening)
Week 8	Only concluding video (~10 min), No practice video	

References

1. Bastien, C.H.; Vallieres, A.; Morin, C.M. Validation of the Insomnia Severity Index as an outcome measure for insomnia research. *Sleep Med* **2001**, *2*, 297-307, doi:10.1016/s1389-9457(00)00065-4.
